# Impact of osteopontin on the development of non‐alcoholic liver disease and related hepatocellular carcinoma

**DOI:** 10.1111/liv.14464

**Published:** 2020-05-30

**Authors:** Alexander D. Nardo, Nicole G. Grün, Maximilian Zeyda, Monika Dumanic, Georg Oberhuber, Elisa Rivelles, Thomas H. Helbich, Daniel F. Markgraf, Michael Roden, Thierry Claudel, Michael Trauner, Thomas M. Stulnig

**Affiliations:** ^1^ Christian Doppler Laboratory for Cardio‐Metabolic Immunotherapy and Clinical Division of Endocrinology and Metabolism Department of Medicine III Medical University of Vienna Vienna Austria; ^2^ Department of Pediatrics and Adolescent Medicine Medical University of Vienna Vienna Austria; ^3^ Division of Nuclear Medicine Department of Biomedical Imaging and Image‐guided Therapy Medical University of Vienna Vienna Austria; ^4^ Department of Pathology General Hospital of Innsbruck Innsbruck Austria; ^5^ Department of Laboratory Medicine Medical University of Vienna Vienna Austria; ^6^ Division of Molecular and Gender Imaging Department of Biomedical Imaging and Image‐guided Therapy Medical University of Vienna Vienna Austria; ^7^ German Diabetes Center Leibniz Center for Diabetes Research Institute for Clinical Diabetology Heinrich Heine University Düsseldorf Germany; ^8^ German Center of Diabetes Research (DZD e.V.) München‐Neuherberg Germany; ^9^ Division of Endocrinology and Diabetology Medical Faculty Heinrich‐Heine University Düsseldorf Germany; ^10^ Hans Popper Laboratory of Molecular Hepatology Division of Gastroenterology & Hepatology Medical University of Vienna Vienna Austria; ^11^ Present address: Hans Popper Laboratory of Molecular Hepatology Division of Gastroenterology & Hepatology Medical University of Vienna Vienna 1090 Austria; ^12^ Present address: Third Department of Medicine and Karl Landsteiner Institute for Metabolic Diseases and Nephrology Hietzing Hospital Vienna 1130 Austria

**Keywords:** acute‐on‐chronic liver failure, fibrosis, lipoapoptosis, metabolic syndrome, non‐alcoholic fatty liver

## Abstract

**Background & aims:**

Osteopontin, a multifunctional protein and inflammatory cytokine, is overexpressed in adipose tissue and liver in obesity and contributes to the induction of adipose tissue inflammation and non‐alcoholic fatty liver (NAFL). Studies performed in both mice and humans also point to a potential role for OPN in malignant transformation and tumour growth. To fully understand the role of OPN on the development of NAFL‐derived hepatocellular carcinoma (HCC), we applied a non‐alcoholic steatohepatitis (NASH)‐HCC mouse model on osteopontin‐deficient (Spp1^−/−^) mice analysing time points of NASH, fibrosis and HCC compared to wild‐type mice.

**Methods:**

Two‐day‐old wild‐type and Spp1^−/−^ mice received a low‐dose streptozotocin injection in order to induce diabetes, and were fed a high‐fat diet starting from week 4. Different cohorts of mice of both genotypes were sacrificed at 8, 12 and 19 weeks of age to evaluate the NASH, fibrosis and HCC phenotypes respectively.

**Results:**

Spp1^−/−^ animals showed enhanced hepatic lipid accumulation and aggravated NASH, as also increased hepatocellular apoptosis and accelerated fibrosis. The worse steatotic and fibrotic phenotypes observed in Spp1^−/−^ mice might be driven by enhanced hepatic fatty acid influx through CD36 overexpression and by a pathological accumulation of specific diacylglycerol species during NAFL. Lack of osteopontin lowered systemic inflammation, prevented HCC progression to less differentiated tumours and improved overall survival.

**Conclusions:**

Lack of osteopontin dissociates NASH‐fibrosis severity from overall survival and HCC malignant transformation in NAFLD, and is therefore a putative therapeutic target only for advanced chronic liver disease.

**Key points**

- Non‐alcoholic fatty liver disease (NAFLD) is currently driving the increase in hepatocellular carcinoma (HCC) prevalence worldwide
- Dysregulated osteopontin (OPN) expression was associated with both obesity‐derived metabolic syndrome and HCC malignant development. However, a causal role for OPN in the pathogenesis of NAFLD and NAFLD‐derived HCC has yet to be established
- In a mouse model of NAFLD‐derived HCC, OPN deficiency worsens hepatic steatosis and fibrosis, while improving systemic inflammation, overall survival and HCC outcomes
- Therefore, we propose OPN as a putative therapeutic target in advanced, metabolic syndrome‐associated chronic liver diseases.

## 1 INTRODUCTION

Hepatocellular carcinoma (HCC) is the most common form of liver cancer and the cause of approximately one million deaths yearly, with an alarming mortality rate of 94%.^1^ Even though hepatitis B‐ (HBV) and C (HCV) virus infections are major risk factors for HCC, nutrition‐related diseases strongly promote the increase in HCC prevalence worldwide. This may be due to a chain of events starting with obesity, metabolic syndrome, type‐2 diabetes mellitus (T2DM) and fatty liver and potentially leading to non‐alcoholic liver steatohepatitis (NASH), liver fibrosis and finally HCC.

Non‐alcoholic fatty liver (NAFL) will likely drive the increase in the incidence of HCC in the next decades.^2^ Approximately 20% of patients manifesting simple steatosis further develop non‐alcoholic steatohepatitis.^3^ NASH mainly occurs when the rate of liver non‐esterified fatty acids (NEFA) uptake surpass its capacity for esterification into triglycerides (TG).^4^, ^5^ NASH, consequently, is a driver for the development of fibrosis/cirrhosis and finally HCC.^6^ Hence, the study of hepatocarcinogenesis on a metabolic syndrome background is gaining significant interest on the individual as well as public health level. However, underlying molecular mechanisms are far from being understood.

Osteopontin (OPN; gene *Spp1*) is a multifunctional protein highly expressed in activated macrophages and T‐cells, but also in hepatic stellate cells and hepatocytes. In obesity, OPN is vastly overexpressed in adipose tissue and induces infiltration and activation of macrophages generating a pro‐inflammatory environment, which crucially contributes to the onset of insulin resistance.^7^ Hepatic OPN expression is up‐regulated in obesity^8^ and various models of liver injury.^9^, ^10^ Furthermore, OPN is involved in the pathogenesis of NAFL associated with visceral obesity ^11^ and is a reliable biomarker for NASH/fibrosis in human non‐alcoholic fatty liver disease (NAFLD).^12^, ^13^ Studies from others and our lab point to a pivotal role of OPN in obesity‐driven nutrition‐dependent diseases including high‐fat diet‐induced fatty liver ^14^, ^15^, ^16^, ^17^ and thus suggest OPN as a treatment target.

Also downstream in the proposed order of events, OPN is highly upregulated in HCC and may even be evaluated as a potential therapeutic target in HCC.^18^, ^19^ However, a causal role of OPN in the pathogenesis of NASH and NASH‐derived HCC is still not defined.To elucidate the impact of OPN on the sequential development of NASH and derived HCC as occurring in metabolic syndrome we took advantage of a novel mouse model,^20^ which starting from hyperglycaemia recapitulates the development of NAFL, NASH, fibrosis up to HCC. Applying this model to wild‐type (WT) and OPN‐deficient (*Spp1* ^−/−^) animals, we provide evidence for a Janus‐type role of OPN in various states of NASH‐HCC progression eventually resulting in enhanced NAFL, NASH and fibrosis but also more highly differentiated HCC and improved overall survival rate in *Spp1* ^−/−^ mice.

## 2 METHODS

### 2.1 Animals

All experimental procedures were approved by the institutional animal care and use committees. Wild‐type and Osteopontin knock out (*Spp1* ^−/−^, B6.129S6(Cg)‐Spp1tm1Blh/J) mice on a C57BL/6J background were purchased from Charles River Laboratories Inc (Wilmington, Massachusetts, USA) and cohoused to minimize potential microbiome effects. Animals were treated as originally described by Fujii and colleagues ^20^: two‐day‐old male newborns of both genotypes received a single subcutaneous STZ (Sigma, Missouri, USA) injection and were fed ad libitum a high‐fat diet (HFD, 60 kcal%, D12492; Research Diets, New Jersey, USA) starting at 4 weeks of age. 14‐hours fasting blood sugar (FBS) and body weight (BDW) were assessed in 4‐week‐old mice, and four FBS‐matched cohorts per genotype were generated (n = 15). Of each group, a representative subgroup of eight animals (FBS‐matched between genotypes) with lower alpha‐diversity was used for molecular analyses. Three‐to‐four littermate mice were housed together in wooden bedding–containing cages in the presence of cage enrichment in a light‐controlled and temperature‐controlled facility. At each time point, animals were overnight fasted (dark cycle) and later sacrificed by neck dislocation (light cycle). To assess in vivo proliferative potential of hepatocytes, all mice received an intraperitoneal injection of 5‐Bromo‐2′‐deoxyuridine (BrdU) (Sigma) 2 hours prior to sacrifice. Blood samples were drawn from the tail vein. Liver, subcutaneous and visceral white adipose tissue (SWAT, GWAT), kidney and small intestine samples were weighted, formalin‐fixed and/or snap‐frozen for further analyses.

### 2.2 Computed tomography

Liver X‐ray computed tomography (Siemens Inveon µCT, by Siemens Medical Solutions, Knoxville, USA) was used to non‐invasively measure number and volume of liver tumours in isoflurane‐anaesthetized mice. Before the µCT X‐ray examination each mouse was administered 100µL of CT contrast medium intravenously (ExiTron™ nano 6000, Miltenyi Biotec GmbH, Germany) to improve the soft tissue contrast. During the examination, the mice were placed on a heated bed and the vital parameters (body temperature and respiratory rate) were constantly monitored and a protective eye ointment was applied. After completion of the study, mice were sacrificed by neck dislocation and tissue samples were harvested as described above.

### 2.3 Histological analyses

Liver sections from the left lobe were formalin‐fixed, paraffin‐embedded and were stained with haematoxylin/eosin, or Sirius red to assess liver fibrosis. Stained sections were evaluated by an expert pathologist, blind to genotype and study period, following the WHO Classification of Tumours of the Digestive System.^21^ Pancreas section was formalin‐fixed, paraffin‐embedded and stained with haematoxylin/eosin to quantitatively evaluate Langerhans islets. Apoptosis was assessed by terminal deoxynucleotidyl transferase‐mediated dUTP‐biotin nick‐end labelling (TUNEL) staining using the TUNEL Andy Fluor™ 488 Apoptosis Detection Kit (Genecopoeia, Maryland, USA) following product instructions. Hepatocyte proliferation was assessed by BrdU immunostatining (Life Technologies, California, USA). Nuclei were counterstained with Hoechst 33 342 and the proportion of TUNEL positive cells was quantified using ImageJ software (National Institute of Health) in an automated fashion.^22^

### 2.4 Hepatic gene expression analyses

RNA was extracted from livers using TRIzol Reagent (Invitrogen, California, USA). Complementary DNA obtained by reverse‐transcription was amplified using the appropriate, commercially available, gene expression assays (Life Technologies, California, USA). Gene expression was normalized to ubiquitin C (Ubc) and analysed by quantitative real‐time RT‐PCR on an ABI Prism 7000 cycler (Life Technologies, California, USA).

### 2.5 Western blotting

Liver samples were homogenized (Precellis 24, Bertin Technologies, France) in RIPA buffer and centrifuged. Supernatants were collected and assayed for protein concentration via BCA Protein Assay Kit (Thermo Fisher Scientific, Massachusetts, USA). Fifteen milligrams of protein/sample were separated by electrophoresis and blotted on nitrocellulose membranes (Bio‐Rad, California, USA), which were then blocked and incubated overnight with a primary antibody to OPN (AF808, R&D Systems, Minnesota, USA) 1:2000 diluted, and beta‐actin (A1978, Sigma, Missouri, USA) 1:10 000. After incubation with secondary antibodies, membranes were developed via the Fusion FX Western Blotting and Chemi Imaging (Vilber Lourmat, France), using the BM Chemiluminescence Blotting Substrate (Roche, Switzerland). Bands intensity was quantified using ImageJ software (National Institute of Health, Maryland, USA).

### 2.6 Plasma biochemistry

Blood glucose was measured using a One Touch Ultra glucose meter (LifeScan Inc, California, USA). Plasma alanine aminotransferase activity (ALT) was determined using the Vitros 5600 technology (Ortho Clinical Diagnostics, New Jersey, USA). Insulin was measured using an ultrasensitive mouse insulin ELISA (Mercodia AB, Sweden), serum amyloid P (SAP) by a commercially available ELISA kit (ALPCO Immunoassays, New Hampshire, USA). NEFA concentration was enzymatically assessed using a commercially available kit (Sigma, Missouri, USA).

### 2.7 Liver biochemistry

TG concentration was enzymatically assessed using a commercially available kit (Sigma, Missouri, USA), after chloroform‐methanol lipid extraction.

### 2.8 Targeted lipidomics of diacylglycerols and ceramides

Lipids were extracted, purified and analysed from frozen samples, using lipid chromatography mass spectrometry as adapted from Kumashiro et al.^23^ Briefly, tissue was homogenized in 20 mM Tris/HCl, 1 mM EDTA 0.25 mM EGTA, pH 7.4, internal standards were added and samples were centrifuged for 1 h (100 000 *g*, 4°C). Lipid droplet, cytosol and membrane fractions were collected and lipids of each fraction were extracted,^24^ followed by solid phase extraction (Sep Pak Diol Cartridges; Waters, Milford, MA, USA). The resulting lipid phase was dried and re‐suspended in methanol. Lipid analytes were separated using a Phenomenex Luna Omega column (1.6 µm 100A; Phenomenex, Torrance, CA, USA) on an Infinity 1290 HPLC system (Agilent Technologies, Santa Clara, CA, USA) and analysed by multiple reaction monitoring on a triplequadrupole mass spectrometer (Agilent 6495; Agilent Technologies), operated in the positive ion mode.

### 2.9 Statistics

Data are presented as mean ± SEM, and two groups compared by unpaired Student t‐test, with a significance level of <0.05. When more than two groups were compared, ANOVA with Tukey´s post hoc analyses were performed. For the survival observations, Kaplan‐Meyer analysis was applied.

## 3 RESULTS

### 3.1 Increased hepatic lipid uptake in NASH‐HCC OPN‐deficient mice promotes liver steatosis

**FIGURE 1:**
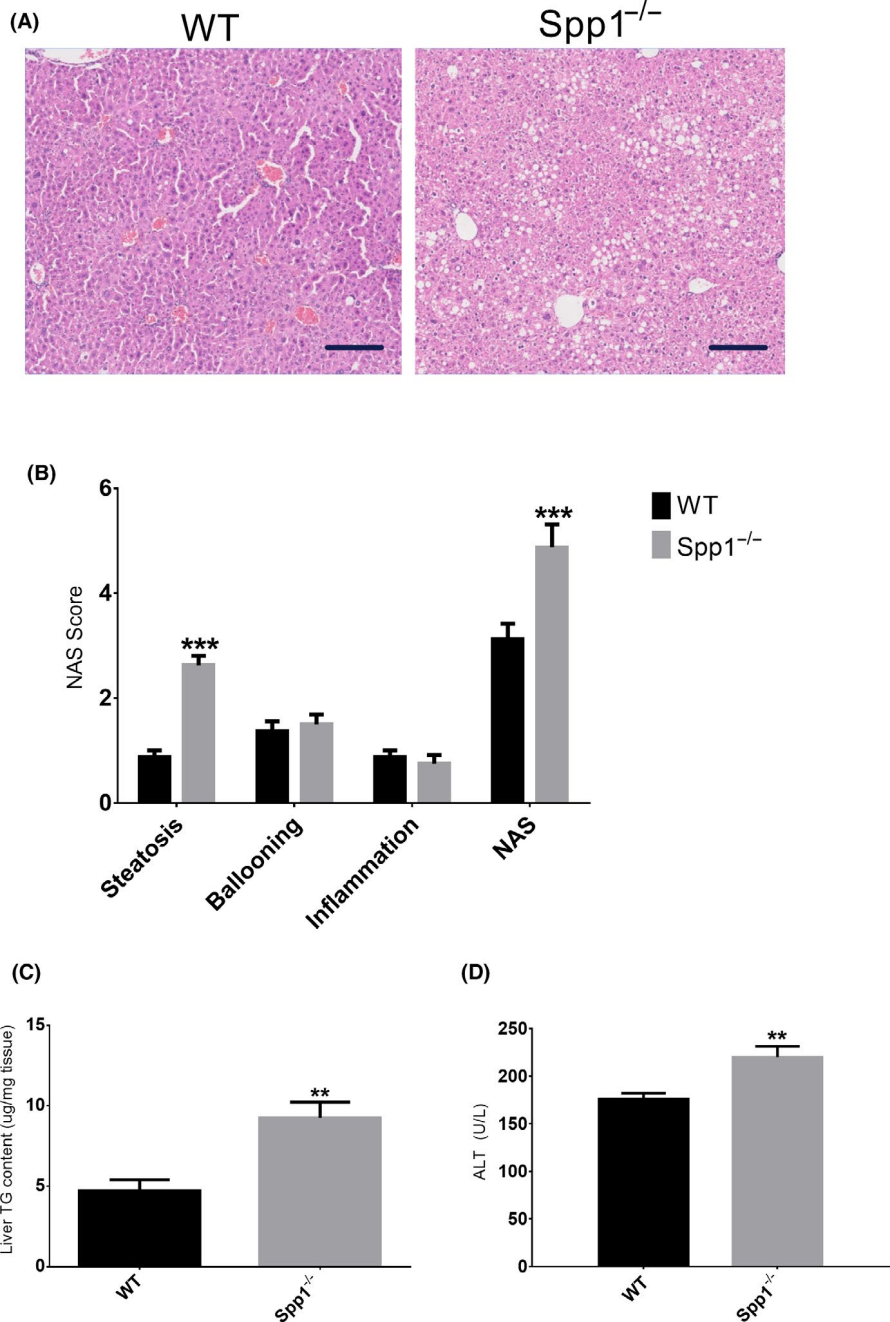
Lack of osteopontin enhances hepatic steatosis in NASH/HCC mice. A, Representative H&E‐stained sections of WT (left panel) and OPN knock‐out (*Spp1* ^−/−^, right panel) NASH‐HCC livers. Scale bar = 100 µm. B, Non‐alcoholic fatty liver disease score (NAS), qualitatively evaluated out of the H&E‐stained sections. NAS is the sum of the scores attributed to the three hallmarks of NAFLD, ie steatosis, hepatocellular ballooning and inflammation. C‐D, Liver triacylglycerol content (C) and plasma alanine aminotransferase activity (D). **P ≤ .01, ***P ≤ .001. Tissues were harvested from 8‐week‐old mice (n = 8 per group). See also Figures S1 and S2

*Spp1* ^−/−^ and WT mice both developed comparable degrees of hyperglycaemia early after streptozotocin (STZ) treatment (plasma glucose 378 ± 16 mg/dL and 348 ± 20 mg/dL, respectively), indicating comparable efficiency of STZ administration. In line with that, the magnitude of STZ‐induced Langerhans islets damage was also comparable between genotypes (Figure S2B). Mice were sacrificed after 4 weeks of high fat diet (HFD) treatment to evaluate NAFL/NASH phenotype. Body weight as well of weights of GWAT and SWAT and plasma NEFA were similar (Figure S1). As shown by haematoxylin and eosin‐staining (Figure 1A) and the NAS score (Figure 1B) as well as hepatic TG quantification (Figure 1C), there was a significantly increased lipid accumulation in livers of *Spp1* ^−/−^ mice. Accordingly, plasma alanine aminotransferase (ALT) was markedly elevated in the *Spp1* ^−/−^ genotype (Figure 1D).

**FIGURE 2:**
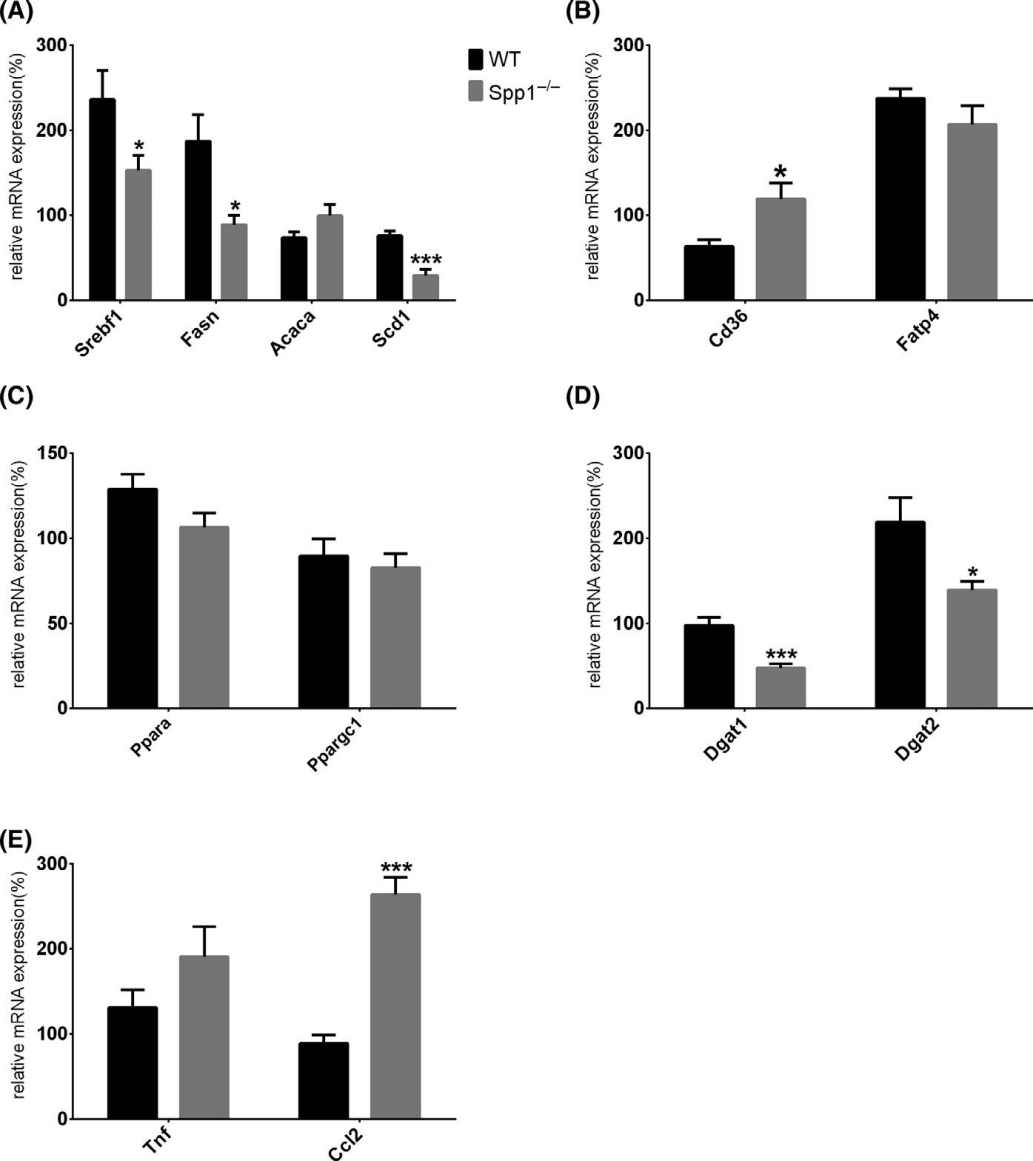
Enhanced hepatic steatosis in *Spp1* ^−/−^ mice might be induced by increased CD36‐mediated lipid uptake. A‐E, Analysis of gene expression regulating de novo lipogenesis (A), hepatic fatty acid uptake (B), beta‐oxidation (C), fatty acid esterification (D) and inflammation (E). *P ≤ .05, ***P ≤ .001. Tissues were harvested from 8‐week‐old mice (n = 8 per group). See also Figure S2

To evaluate the potential mechanisms of hepatic TG accumulation in *Spp1* ^−/−^ animals, we measured the expression of genes playing a pivotal role in hepatic lipid homeostasis. Surprisingly, expression of *Acaca* was comparable between WT and *Spp1* ^−/−^ mice, while *Srebf1*, *Fasn* and *Scd1* where even significantly downregulated in the *Spp1* ^−/−^ genotype (Figure 2A), indicating that the increased steatosis observed in *Spp1* ^−/−^ mice cannot be explained by enhanced de novo lipogenesis (dnl). Also, expression levels of *Ppara* and *Ppargc1a* did not differ between the two groups (Figure 2C), while Dgat1 and Dgat2 enzymes were markedly downregulated in the *Spp1* ^−/−^ group (Figure 2D). While the gene for the fatty acid transporter FATP4 (*Slc27a4*) was unchanged between genotypes (Figure 2B), gene expression of fatty acid translocase (*CD36/FAT*), a member of the class B scavenger receptor family essential for fatty acid (FA) uptake and lipid metabolism, was markedly increased in *Spp1* ^−/−^ compared to WT animals (Figure 2B). Also, while inflammation was evaluated as comparable between groups by liver histology (Figure 1B), gene expression analyses showed a non‐significant and significant increase in expression of the main hepatic inflammatory markers *Tnf* and *Ccl2* in *Spp1* ^−/−^ mice respectively (Figure 2E). These data strongly emphasize that increased liver lipid uptake by overexpression of the CD36 FA translocase could contribute to the increased hepatic steatosis and inflammation in NASH‐HCC‐*Spp1* ^−/−^ animals. These differences are observable only upon metabolic challenge (Figure S2A), meaning that the lack of OPN expression does not influence hepatic lipid metabolism at baseline.

### 3.2 Increased liver fibrosis in OPN‐deficient mice

**FIGURE 3:**
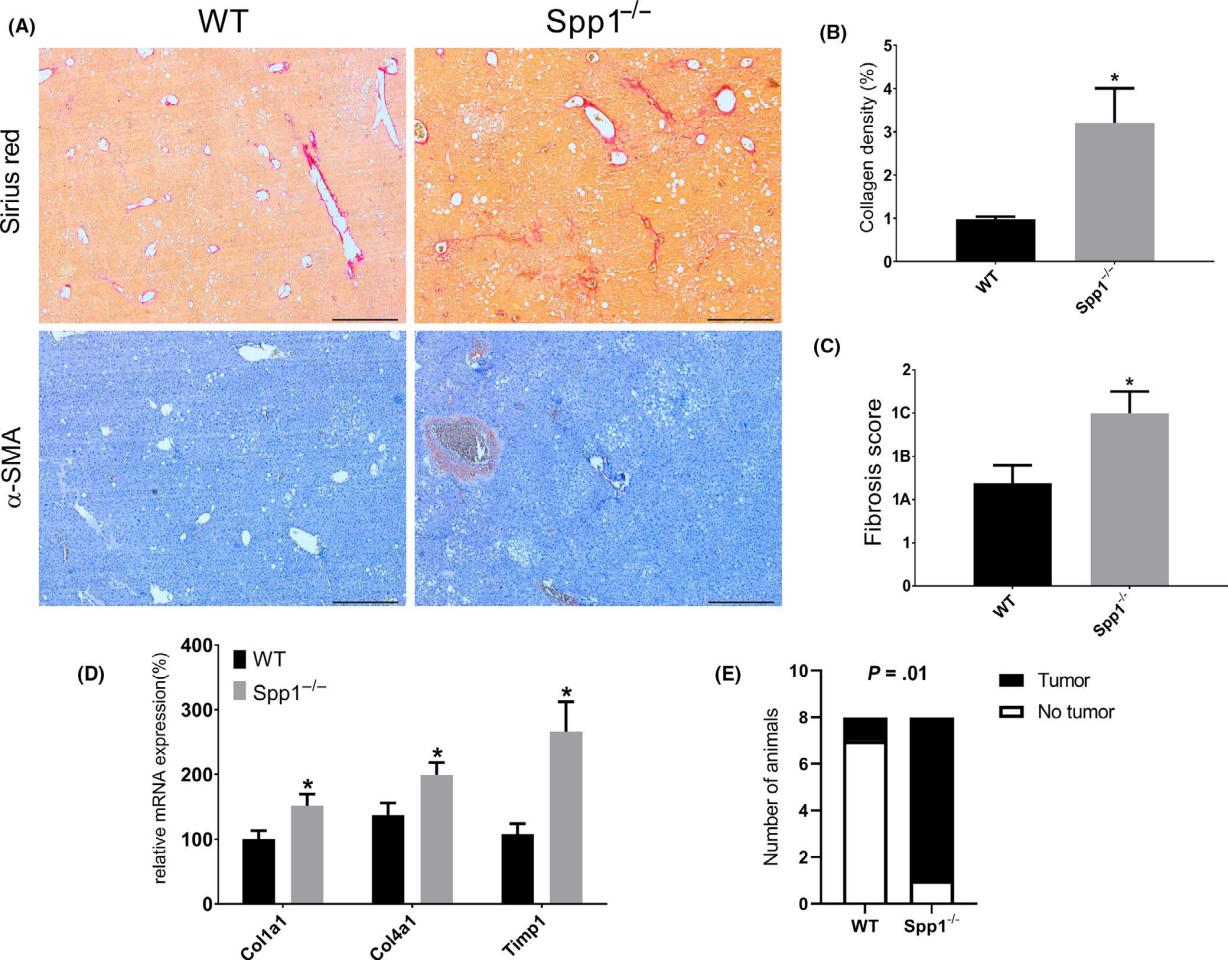
OPN protects against NAFL/NASH‐induced hepatic fibrosis. A, Representative Sirius red (upper panels) and α‐SMA (lower panels) stained sections of WT (left panels) and *Spp1* ^−/−^ (right panels) NASH‐HCC livers. Scale bar = 500 µm. B, Collagen deposition (red signal) was quantified in an automated fashion. C, Fibrosis score, qualitatively assessed on H&E‐stained liver sections by an expert pathologist, blind to the genotype. D, Gene expression analysis of the main pro‐fibrogenic markers. E, Tumour incidence at the fibrosis stage (week 12). *P ≤ .05. Tissues were harvested from 12‐week‐old mice (n = 8 per group)

**FIGURE 4:**
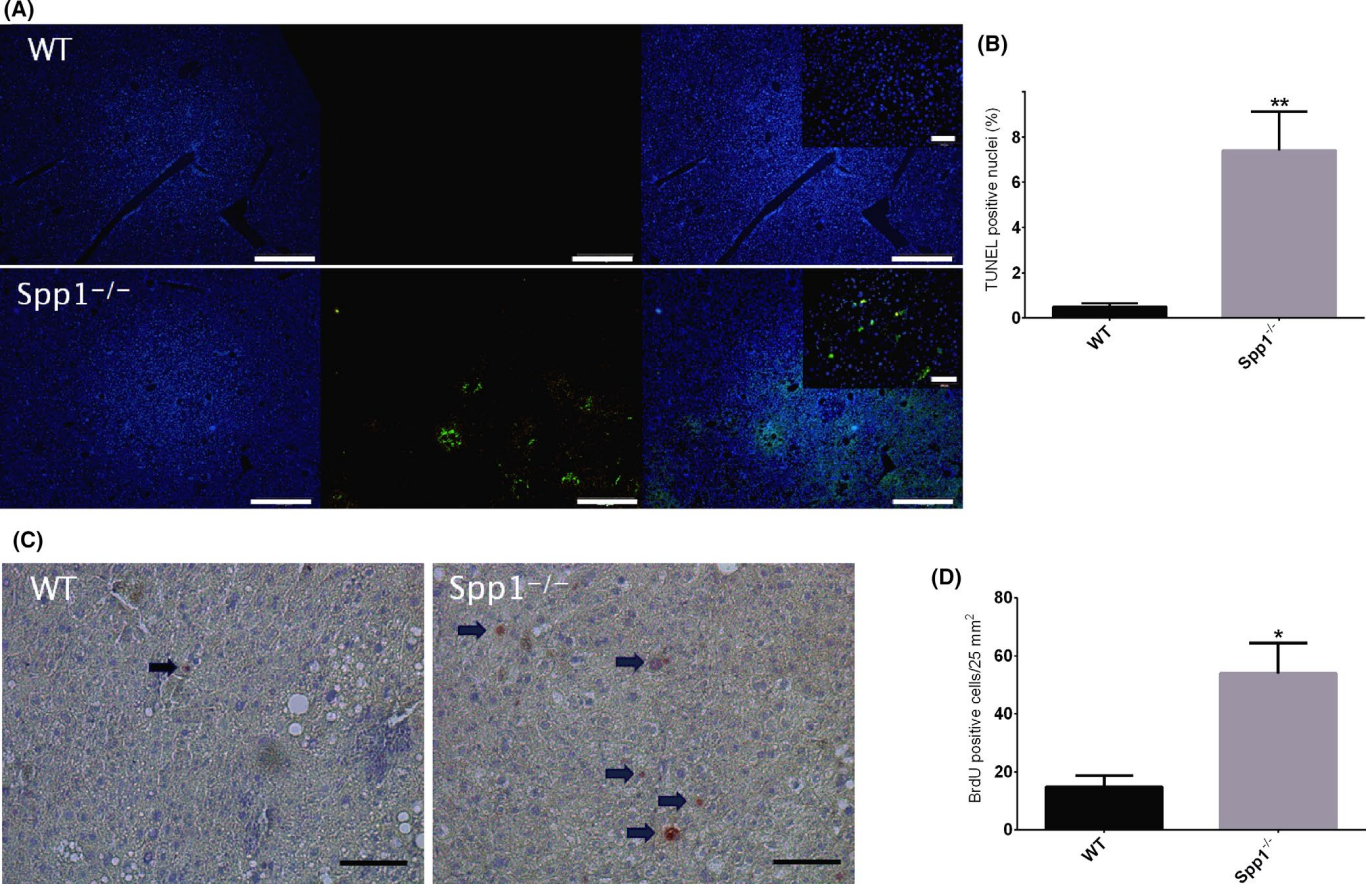
Enhanced apoptosis and proliferation in *Spp1* ^−/−^ mice. A, TUNEL assay for the identification of apoptotic cells in WT (upper panel) and *Spp1* ^−/−^ (lower panel) NASH‐HCC livers. Hoechst 33 342 (blue signal, left panels) stains all nuclei, while apoptotic cells are marked with green fluorescence (central panels). Right panels represent the merge of the two signals. Scale bar = 200 µm. Inserts at higher magnification, scale bar 50 µm. B, Graph of the proportion of TUNEL‐positive cells. C, BrdU staining for the identification of proliferating cells. Sections were incubated with anti‐BrdU antibody and counterstained with haematoxylin. Positive nuclei are depicted in red and highlighted by black arrows. Scale bar = 50 µm. *P ≤ .05, **P ≤ .01. D, Analysis of BrdU‐positive cells/25 mm^2^ area. Tissues were harvested from 12‐week‐old mice (n = 8 per group)

Hepatic fibrosis evolves from NAFL/NASH on a metabolic syndrome background. In line with the worse steatotic phenotype of *Spp1* ^−/−^ livers, markedly increased liver collagen deposition and myofibroblast activation were observed in *Spp1* ^−/−^ mice as shown by Sirius Red staining and α‐SMA immunostaining respectively (Figure 3A,B). While WT mice just showed mild‐to‐moderate perisinusoidal and zone‐3 fibrosis, fibrosis was also extended to the portal and periportal area resulting in a higher fibrosis score in *Spp1* ^−/−^ livers (Figure 3C). Only these livers also showed well‐defined bridging fibrosis and fibrotic septa (Figure 3A). In addition, a significant increase in gene expression of pro‐fibrogenic markers (*Col1a1, Col 4a1, Timp1*) was found in *Spp1* ^−/−^ mice (Figure 3D). Of note, no changes in *Tgfb* expression were observed between genotypes and time points (Figure 7C). Hepatocellular apoptosis is implicated in the progression of fibrotic liver disease.^25^ Since we and others have previously shown the anti‐apoptotic potential of OPN,^15^, ^26^ we hypothesized that enhanced apoptosis in OPN‐deficient livers could contribute to the increased hepatic fibrosis. Accordingly, terminal deoxynucleotidyl transferase‐mediated dUTP‐biotin nick‐end labelling (TUNEL) staining of liver sections revealed an increased proportion of apoptotic cells in OPN‐deficient mice compared to WT (Figure 4A,B). To assess hepatocyte proliferation, the number of hepatocytes in replicative S‐phase was evaluated by BrdU immunostaining. The percentage of proliferating hepatocytes was significantly higher in the livers of *Spp1* ^−/−^ mice (Figure 4C,D), thus indicating a compensatory hyperproliferation of hepatocytes.

### 3.3 Accumulation of specific diacylglycerol species during NAFL in NASH‐HCC‐*Spp1* ^−/−^ mice

**FIGURE 5:**
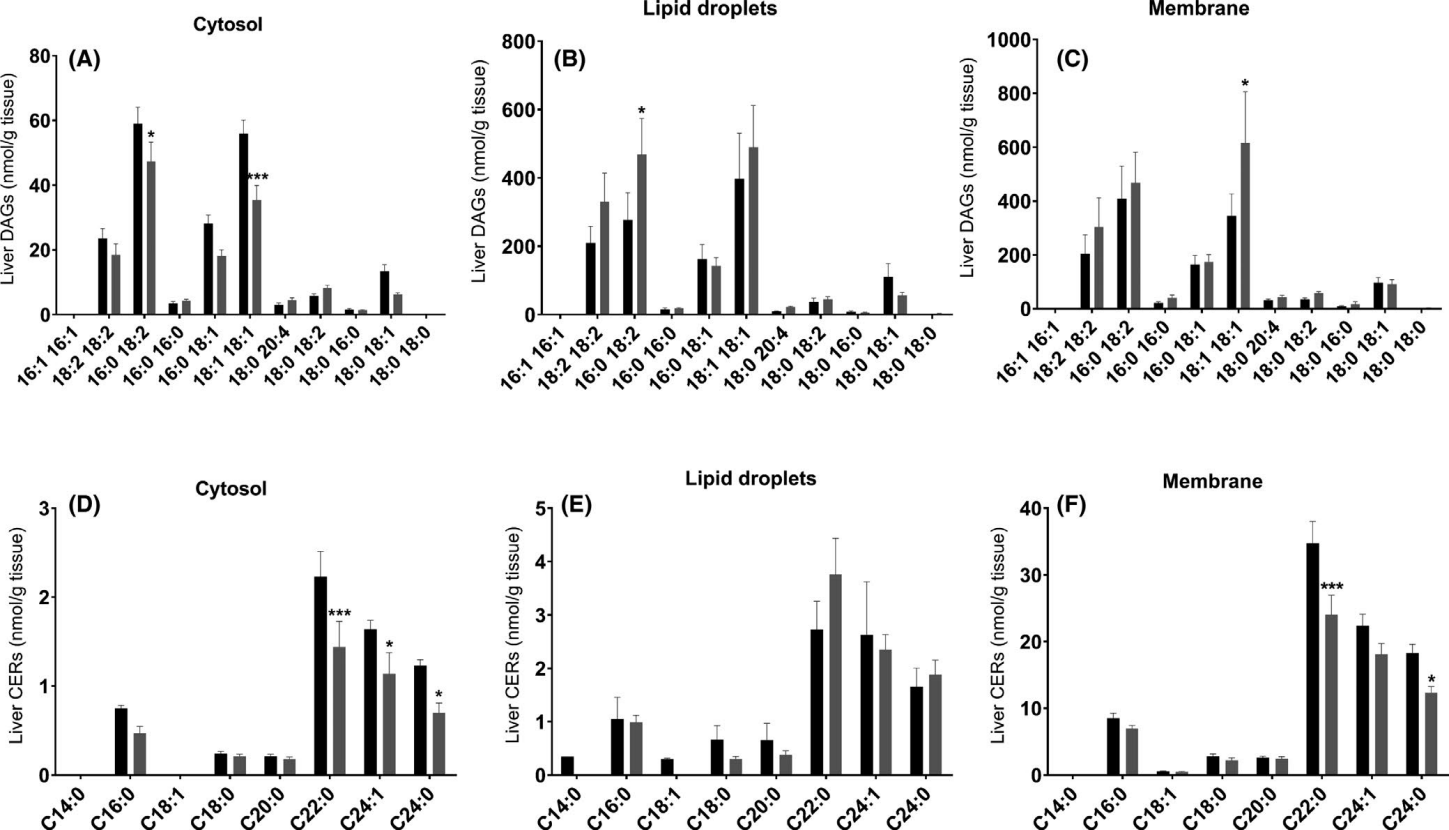
Targeted lipidomic analyses at the NAFL/NASH time point. A‐C, Total amount of individual DAG species in the (A) cytosolic, (B) lipid droplet and (C) membrane fraction. D‐F, Total amount of each individual CER species per cellular fraction. Black bars represent WT, while grey bars *Spp1* ^−/−^ animals. *P ≤ .05, **P ≤ .01, ***P ≤ .001. Tissues were harvested from 8‐week‐old mice (n = 8 per group)

Hepatic lipid accumulation in NASH‐HCC WT and *Spp1* ^−/−^ mice seemed to rely on different mechanisms: increased lipogenesis in NASH‐HCC‐WT, but increased lipid uptake in NASH‐HCC‐*Spp1* ^−/−^ (Figure 2). Looking for potential molecular drivers of the enhanced steatosis and fibrosis developed by NASH‐HCC‐*Spp1* ^−/−^ mice, we performed targeted lipidomic analyses with emphasis on diacylglycerols (DAGs) and ceramides (CERs) of livers at the NAFL/NASH time point. Most of the DAG species were unchanged between genotypes. Membrane DAG 18:1/18:1 significantly increased in the absence of OPN (Figure 5C), as also DAG 16:0/18:2 in the lipid droplet compartment (Figure 5B). Membrane CER 24:0 and especially 22:0 decreased in *Spp1* ^−/−^ livers (Figure 5F). No significant increase in other ceramide species was observed in *Spp1* ^−/−^ when compared with WT mice (Figure 5D‐F). These data support the hypothesis of a putatively lipotoxic accumulation of specific DAG species driving the progression of hepatic disease in NASH‐HCC‐*Spp1* ^−/−^ mice.

### 3.4 Osteopontin deficiency prevents HCC dedifferentiation

Already at 12 weeks of age, which corresponds to the liver fibrotic stage, 87.5% of OPN‐deficient animals developed liver tumours, which were histologically defined as HCCs (data not shown). In the WT group, just 1/8 of the mice showed HCCs at this time point (Figure 3E).

**FIGURE 6:**
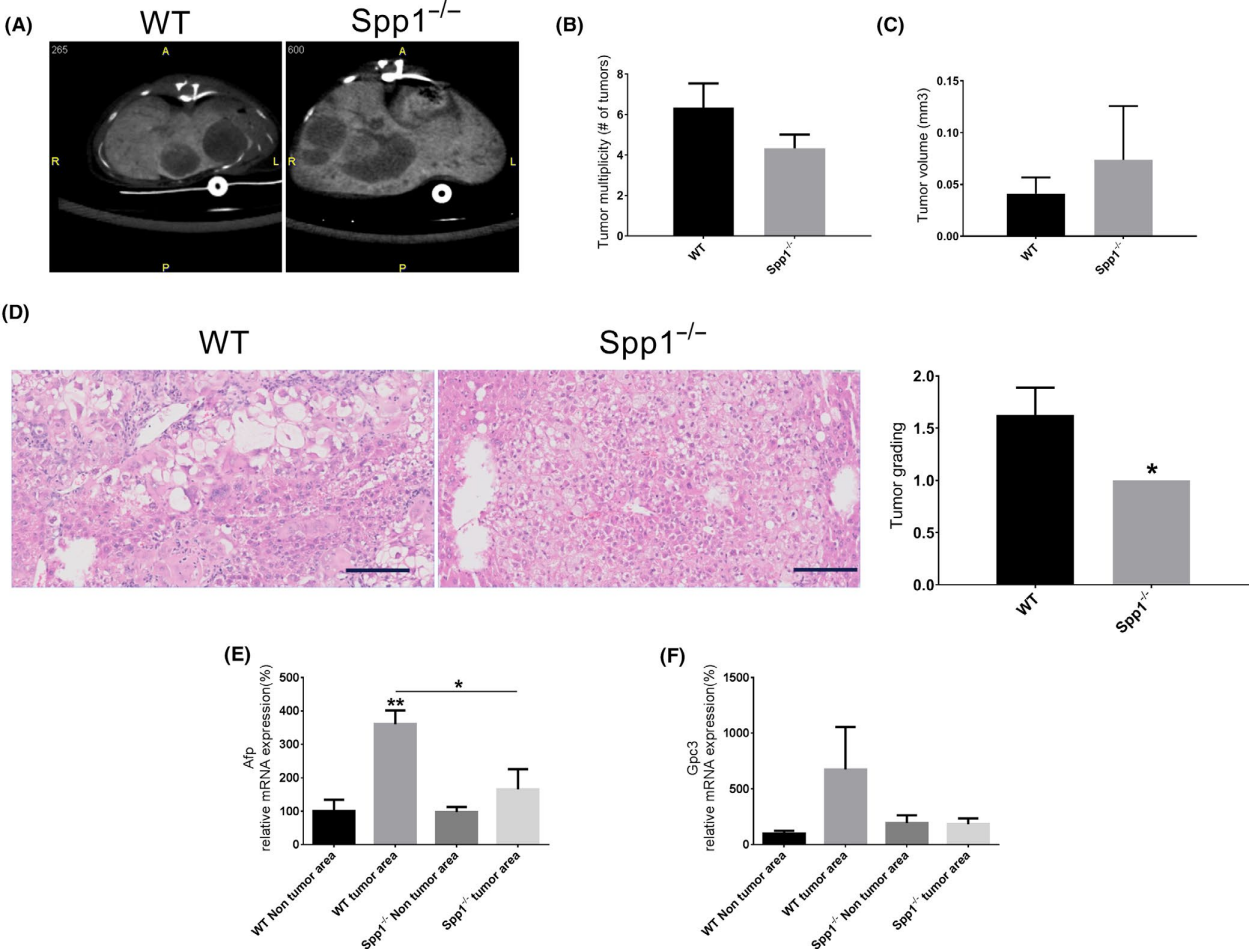
Lack of OPN prevents HCC dedifferentiation. A, Representative in vivo liver X‐ray computed tomography (CT) sections from WT (left panel) and *Spp1* ^−/−^ (right panel) NASH‐HCC mice. B, Number of tumours per liver and (C) tumour volumes were quantitatively evaluated in an automated fashion. D, Tumour grading was histologically evaluated on H&E‐stained liver sections by an expert pathologist, blind to the genotype. E‐F, Gene expression analysis of the HCC markers *Afp* (E) and *Gpc‐3* (F) in both tumour and non‐tumour areas. Tissues were harvested from 19‐week‐old mice. n = 3 per group for CT analyses, otherwise n = 8 per group. **P ≤ .01 compared to WT non‐tumour area

**FIGURE 7:**
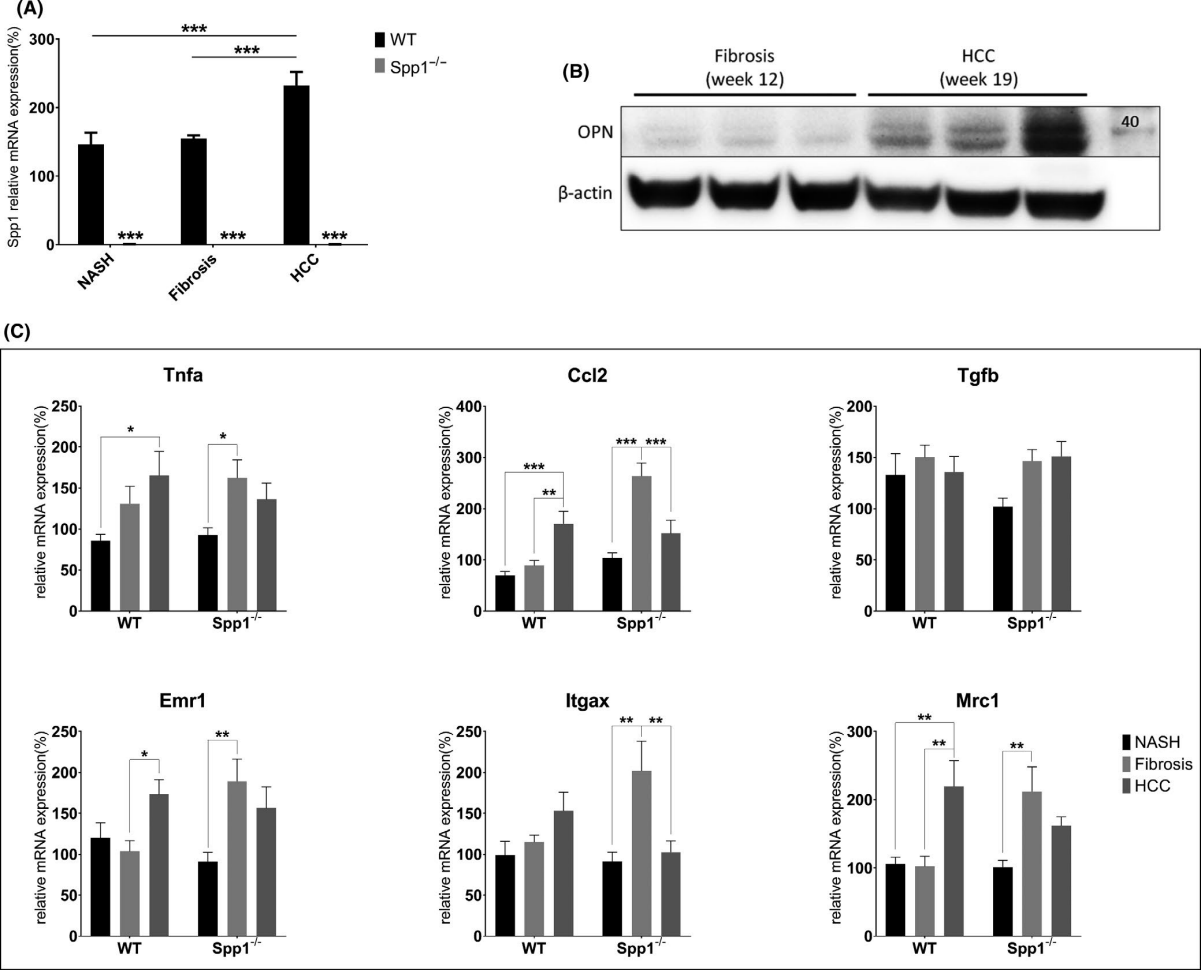
Expression patterns of OPN and other immunological markers. A, Gene expression assessment of OPN, black bars represent WT, while grey bars *Spp1* ^−/−^ animals (n = 8 per group). B, Immunoblot against OPN at different time points in WT animals. C, Hepatic gene expression analyses of *Tnfa, Ccl2, Tgfb, Emr1, Itgax and Mrc1* at 8, 12 and 19 weeks. *P ≤ .05, **P ≤ .01. Tissues were harvested between the 8th and the 19th experimental week. (n = 8 per group)

Mice were finally analysed at 19 weeks of age in order to evaluate hepatocellular carcinomas. CT scans showed widespread liver tumours in both WT and *Spp1* ^−/−^ mice, but no significant differences in tumour size and number were observed (Figure 6A‐C). However, a higher degree of tumour dedifferentiation in WT compared to *Spp1* ^−/−^ animals was shown in histologic analyses, as indicated by a significantly higher tumour grade in livers of wild‐type mice (Figure 6D). Expression levels of HCC markers revealed a marked overexpression of *Afp* in tumours from WT compared to *Spp1* ^−/−^ animals. Moreover when compared with the adjacent non‐tumour tissue, its expression was upregulated only in WT but not in *Spp1* ^−/−^ mice (Figure 6E). mRNA levels of *Gpc‐3* followed the same trend but did not reach statistical significance (Figure 6F). The hampered HCC progression observed in OPN‐deficient mice and the specific overexpression of OPN at the HCC time point in livers of NASH‐HCC‐WT animals (Figure 7A,B) point toward a causative role for OPN in the dedifferentiation of HCCs harboured on a metabolic syndrome‐background.

Given the central role played by OPN in hepatic inflammation and carcinogenesis,^27^, ^28^ and since the immunological milieu significantly influences tumorigenesis,^29^ we further analysed the expression pattern of the principal inflammatory markers during disease evolution. *Tnfa* and *Ccl2* expression peaks at the fibrosis stage in *Spp1* ^−/−^ livers, decreasing again later at the HCC time point (Figure 7C). Of note, the expression level of the same genes increases over time in livers of WT mice and peaks at the HCC stage (Figure 7C). The expression pattern of the pan‐macrophage marker *Emr1* follows the same trends, indicating altered macrophage recruitment in livers of *Spp1* ^−/−^ compared to WT NASH‐HCC mice (Figure 7C). Furthermore, we measured early and transient upregulation of both specific pro‐inflammatory and anti‐inflammatory macrophage markers (*Itgax* and *Mrc1*, respectively) in OPN‐deficient livers. Only *Mrc1*, an M2‐like macrophage marker, is significantly overexpressed at the HCC stage in WT livers (Figure 7C). These data strongly suggest that the lack of OPN hampers HCC progression and dedifferentiation by modulating the hepatic inflammatory kinetics.

### 3.5 Osteopontin deficiency reduces liver‐related mortality

**FIGURE 8:**
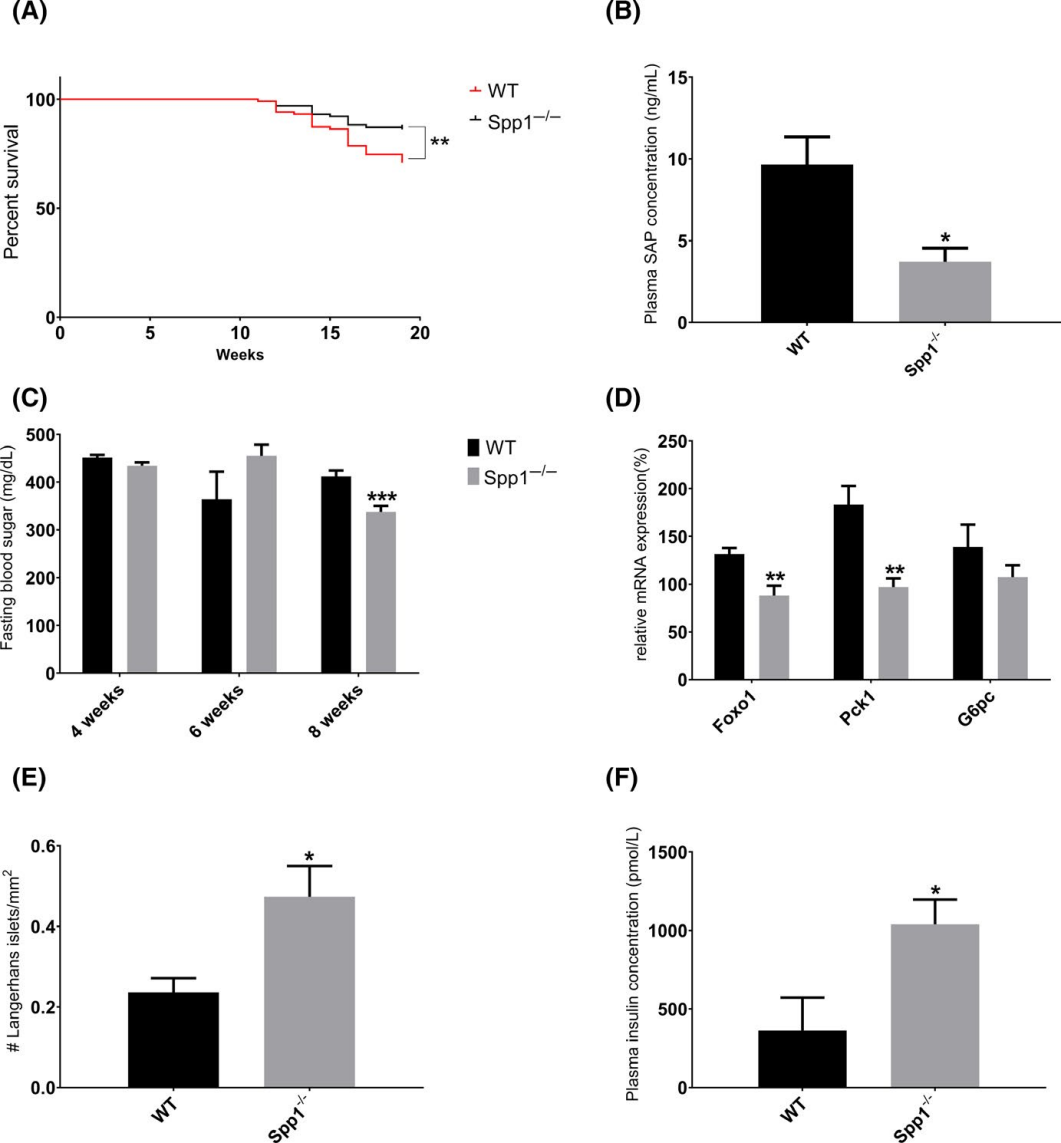
Spp1^−/−^ mice show a healthier overall metabolic homeostasis and an improved survival rate despite worse steatotic and fibrotic liver manifestations. A, Kaplan‐Meyer curve showing survival of WT (red full line) and *Spp1* ^−/−^ (black full line) mice. B, Serum amyloid P (SAP) concentration in the plasma of 8‐week‐old NASH‐HCC mice. C, Fasting blood sugar (FBS) measured during the experimental week 4, 6 and 8. Black bars represent WT, while grey bars Spp1^−/−^ animals. D, Gene expression analysis of the main genes regulating hepatic gluconeogenesis. E, Amount of intact pancreatic Langerhans islets, manually counted on H&E‐stained, scanned sections. F, Enzymatic assessment of plasma insulin levels. *P ≤ .05, **P ≤ .01, *** *P* ≤ .001. n = 15 for metabolic assessments, n = 8 for molecular analyses. See also Figure S3

As described earlier, NASH‐HCC mice started to die after week 11, which corresponds to the liver pre‐fibrotic/fibrotic stage.^30^ In the present study, the mortality after 19 weeks in the WT group was 30% and significantly higher than in *Spp1* ^−/−^ mice (14%; *P* = .0085) (Figure 8A). All dropouts showed symptoms of hepatic toxicity, such as microvesicular steatosis (Figure S3) indicating acute‐on‐chronic liver failure (ACLF) as described in patients with chronic liver disease.^31^ Mortality in clinical ACLF correlates with the magnitude of systemic inflammation,^31^ which was significantly enhanced also in NASH‐HCC‐WT mice as shown by increased serum amyloid P (SAP) levels (Figure 8B). Hence, OPN promotes a systemic pro‐inflammatory milieu, which significantly reduces survival in non‐alcoholic fatty liver disease.

### 3.6 The worse liver condition in OPN‐deficient mice is the consequence of a better overall metabolic homeostasis

NAFLD in metabolic syndrome is related to systemic metabolic dysregulations. We wanted, therefore, to clarify the role of OPN on metabolic homeostasis. At variance with Spp1^−/−^ mice, some NASH‐HCC‐WT animals at eight weeks of age (3 out of 8) showed hepatic cytoplasmic and nuclear deposition of glycogen, symptom of worse glycaemic control. STZ‐treated mice exhibit hyperglycaemia throughout their entire life without differences in fasting blood sugar (FBS) between WT and Spp1^−/−^ mice at 4 and 6 weeks of age (Figure 8C). However, OPN deficiency was associated with slightly though significantly reduced FBS (Figure 8C) and also with higher numbers of intact Langerhans islets (Figure 8E) and increased plasma insulin levels (Figure 8F) in 8‐week‐old NASH‐HCC mice. Moreover mRNA expression of FOXO1 was markedly reduced in NASH‐HCC‐Spp1^−/−^ livers (Figure 8D), and G6pc and Pck1 genes, which encode key gluconeogenic enzymes and are transcriptional targets of FOXO1, were suppressed in OPN‐deficient livers (Figure 8D, statistically significant only for G6pc). Hence, higher insulin secretion and downregulation of hepatic FOXO1 and its target genes in NASH‐HCC Spp1^−/−^ mice could contribute to the beneficial effects of OPN deficiency on glucose metabolism, as also to the detrimental effect on hepatic lipid accumulation and toxicity.

## 4 DISCUSSION

NAFL is a hallmark of metabolic syndrome, the committing step for the potential further evolution of NASH, fibrosis and liver cancer, and the forecasted future leading aetiology for HCC. Insights into the establishment and development of such a sequela of pathophysiological events may provide new interventional strategies focused on blocking, or at least delaying, this yet uncontrollable process. Unfortunately, the impossibility to sample patients´ livers at each stage of the disease and the lack of an animal model that faithfully mirrors the human pathological progression and aetiology made it impracticable until today. In the current study, we used a recently described mouse model, which sequentially develops NASH, fibrosis and HCC on a background of diabetes and obesogenic diet, to finally assess the role of OPN, a putative prognostic and therapeutic target for liver cancer, on the development of HCC in NAFLD. In spite of OPN’s well‐established inflammatory role, absence of OPN even worsened hepatic steatosis and fibrosis, but reduced dedifferentiation of HCCs and liver‐failure‐related mortality in mice on a background of hyperglycaemia and high‐fat diet. Even though OPN exhibits some beneficial effects in early NAFLD stages, it promotes dedifferentiation of HCC and organ failure probably through its pro‐inflammatory function. Most importantly, OPN ablation dissociates NASH‐fibrosis severity from overall survival and HCC malignant transformation. This study hence provides a basis for further translational research to target OPN in order to reduce liver‐related mortality in advanced NAFLD.

Deregulated hepatic NEFA influx covers a predominant role in human NAFLD.^32^, ^33^ An increased hepatic NEFA influx through specific membrane translocases, such as CD36, eventually induces enhanced hepatic steatosis also in the NASH‐HCC mouse model in use, upon genetic OPN deletion. Both experimental and clinical data show that *CD36* expression positively correlates with liver fat deposition under conditions of elevated lipids and induces lipoapoptosis‐dependent inflammation and fibrosis.^34^, ^35^ Elevated lipid uptake is not excluded by the downregulation of hepatic *Dgat1* and *Dgat2* expression. Recently published data demonstrated that substrate flux, and not *Dgat* expression, is the dominant regulator of hepatic TG synthesis.^36^ Moreover the concomitant increment in lipid uptake and reduction in FA esterification potential also explains the significant accumulation of DAGs in *Spp1* ^−/−^ livers. CD36 expression is controlled by peroxisome proliferator‐activated receptor gamma (PPARγ), and the accumulation of linoleic acid, a known source of PPARγ activating ligands,^37^ might explain the significant *Cd36* overexpression in *Spp1* ^−/−^ hepatocytes. Furthermore, OPN was shown to positively impact on JNK downstream pathways,^38^ and PPARγ activity can be inhibited by JNK through phosphorylation of Serine 84.^39^ Therefore, lack of OPN might reduce JNK‐mediated suppression of PPARγ, resulting in increased *Cd36* expression. In a considerably less hyperglycaemic model, which does not develop further fibrosis and HCC, it has been shown that lack of OPN protects from HFD‐induced hepatic steatosis, due to the preservation of adipose tissue function in obesity, hence preventing ectopic lipid accumulation in the liver.^16^, ^17^ In contrast, the model used here is primarily hyperglycaemic but not obese hence revealing the impact of hyperglycaemia in this process while excluding a potential involvement of a dysfunctioning adipose tissue.

Several experimental and clinical studies also indicate that OPN is involved in liver fibrogenesis.^40^, ^41^ However, previous research did not use models that replicate clinicopathological features of NAFL‐related fibrosis. In our model, hepatic fibrogenesis evolves from NAFL/NASH on a diabetes background. The lack of OPN not only worsened the steatotic and inflammatory phenotypes, but also enhanced hepatic collagen deposition in NASH‐HCC‐*Spp1* ^−/−^ mice. We also provide evidence of enhanced apoptosis and proliferation rate in *Spp1* ^−/−^ livers, phenomena that tightly correlate with exaggerated tissue repair and fibrogenesis. Overexpression of *Timp1*, a known inhibitor of the elastin‐degrading MMP12 and marker of tardive fibrosis,^42^ further contributes to the more advanced fibrosis in *Spp1* ^−/−^ livers. While OPN ablation was shown to reduce leptin‐induced hepatic fibrosis in vitro* and* in vivo,^40^ and ductal reaction and fibrosis in thioacetamide (TAA)‐treated mice,^43^ the NASH‐HCC model, which closely mirrors the full development of human disease up to HCC, reveals that NASH‐fibrosis may even exaggerate in the absence of OPN, when hyperglycaemia and lipotoxicity are the driving forces.

Specific lipid metabolites are known to contribute to NASH‐HCC development. The significant accumulation of DAGs could be a cue for enhanced lipotoxicity‐induced hepatocellular apoptosis in NASH‐HCC‐*Spp1* ^−/−^ animals. The significant enrichment in DAG species containing palmitic and linoleic acid in lipid droplets further confirms the paramount effect of FA uptake as mechanism of increased steatosis in NASH‐HCC‐*Spp1* ^−/−^ mice. The DAG and CER profiles obtained are in line with previous studies ^44^, ^45^ and confirm again the worse non‐alcoholic fatty liver disease phenotype developed in the absence of OPN. To pinpoint the role of lipotoxicity on tissue‐specific and systemic inflammation, which is proposed by us as a key player in mortality and HCC dedifferentiation, we plan to perform direct measures of FA uptake by the liver and a detailed quantification of extrahepatic lipid distribution.

At 12 weeks of age, NASH‐HCC‐*Spp1* ^−/−^ mice harboured significantly more spontaneous HCCs than WT. Previous reports on NASH‐HCC‐WT animals just identified simple hepatic nodules at this time point,^20^, ^30^ suggesting OPN as a key tumour suppressor factor in early HCC development. The increased proliferation rate probably compensating for enhanced apoptosis may have paved the way to early HCC development in *Spp1* ^−/−^ mice. Interestingly, at 19 weeks of age, no significant differences in tumour size and number between genotypes were measured. However, tumours harboured in OPN‐expressing, WT animals reached a significantly higher level of dedifferentiation compared with HCCs grown in OPN‐deficient livers, which remained well‐differentiated. The specific overexpression of OPN at the HCC time point in livers of NASH‐HCC‐WT animals may hence be related to dedifferentiation of HCCs, and consequently increase tumour and metastasis‐related risks. Our data are in line with previous publications asserting that OPN mainly promotes late events in hepatocarcinogenesis, such as epithelial‐to‐mesenchymal transition (EMT), invasion and metastasis.^46^, ^47^ Based on our results, we also provide evidence that OPN induces HCC progression and dedifferentiation by modulating the hepatic inflammatory kinetics. The significant overexpression of pro‐inflammatory cytokines during the fibrosis time point seems to induce macrophage recruitment into the livers of *Spp1* ^−/−^ mice. As shown by the relative expression of the main macrophage polarization markers, a comparable amount of them are M1‐ and M2‐activated, which may elicit anti‐tumour processes. At the HCC time point, this recruitment is significantly blunted in OPN‐deficient livers, indicating a possible resolution, or at least a good control of tumour development. In WT mice, on the other hand, significant overexpression of pro‐inflammatory cytokines occurs later in time, at the HCC time point in parallel with macrophage recruitment, at a time when OPN is also strongly overexpressed. The selective overexpression of the M2‐like macrophage polarization marker Mrc1 let us presuppose an accumulation of tumour‐associated macrophages (TAMs) ^48^ in NASH‐HCC WT livers. Hence, the adverse hepatic immunological milieu in later HCC development correlates with OPN expression, indicating again its detrimental role in the malignant outcome of liver cancers.

Even though OPN deficiency in NASH‐HCC mice induces stronger steatosis, steatohepatitis and fibrosis, it significantly protects against organ failure‐related death, which closely resembles human acute‐on‐chronic liver failure. Indeed, the development of acute on chronic liver failure occurs in the setting of systemic inflammation, the severity of which correlates with the number of organ failures and mortality.^31^ As shown by plasma SAP levels of NASH‐HCC mice at the NAFL/NASH time point, systemic inflammation is dramatically higher in WT mice, most probably because of the ectopic lipid deposition and the consequent lipotoxicity and failure of non‐metabolic organs. Hence, OPN probably promotes liver failure due to its pro‐inflammatory action.

The liver has a fundamental role in maintaining hepatic and whole body glucose and lipid homeostasis: it senses these metabolic moieties and a plethora of hormones and other molecular mediators, integrates the signals and responds providing the right balance between glucose and lipid uptake, synthesis, storage and secretion. Our investigations show that lack of OPN protects against the HFD‐induced pancreatic lipotoxicity and improves therefore the overall metabolism and the hepatic function in Spp1^−/−^ mice. Indeed, we showed that lack of OPN protects against cytoplasmic and nuclear glycogen deposition in hepatocytes. Since hepatocellular glycogen deposition is the principal clinical manifestation of glycogenic hepatopathy in humans,^49^ we further investigated glycaemic control in vivo, which was improved in OPN‐deficient animals. Moreover the fact that fasting blood sugar showed significant improvement only after four weeks of HFD and that the amount of intact Langerhans islets was comparable between genotypes before the HFD challenge suggests that OPN deficiency did not interfere with initial STZ treatment but improved beta‐cell survival or recovery thereafter, maybe by reduced lipotoxicity.^50^, ^51^ On the other hand, upon sustained hyperglycaemia and hyperlipidaemia, the more responsive OPN‐deficient liver gets more severely damaged by the lipid overload, while protecting other organs, such as pancreas and possibly skeletal muscles, and preventing therefore systemic inflammation and mortality. On the contrary of OPN neutralizing studies by means of anti‐OPN antibodies,^15^, ^19^, ^52^ completely depleting OPN action disrupts the paradigm that a more severe NASH phenotype will increase the risk of severe fibrosis development and in turn also liver‐related mortality.^53^, ^54^, ^55^, ^56^ Hence our study emphasizes a central role for osteopontin in NAFLD development and might guide future studies on therapeutic interventions based on this multifunctional cytokine.

In summary, on a hyperglycaemic background resembling insufficiently controlled diabetes, OPN exerts not only detrimental but also beneficial roles with respect to the development of NASH and progression to HCC. OPN is likely involved in the intrinsic control of excessive lipid uptake by the liver, and hence protects from lipotoxicity, apoptosis and consequent fibrosis and hepatocyte proliferation, which leads to differentiated HCC. On the other hand, OPN promotes metabolic dysregulations observed in metabolic syndrome, dedifferentiation of HCC and organ failure probably through its pro‐inflammatory function. This study hence demonstrates a bimodal Janus‐type action of OPN, being tumour‐protective at the early stage while tumorigenic in the progressive phase. Furthermore, OPN most likely plays a systemic role on inflammation and a hepatic role on HCC malignant transformation in NAFLD. Now as the roles of OPN in NASH‐HCC development are established, further research will focus on molecular mechanism of OPN action in early *vs*. late stages of this devastating disease in order to elucidate potential strategies for its prevention or treatment.

## DECLARATION OF INTEREST

The authors declare no competing interests.

## Supporting information

Supplementary Material
