# Supplementary material for: Impact of osteopontin on the development of non‐alcoholic liver disease and related hepatocellular carcinoma
Source: Liver Int. 2020 May 30;40(7):1620–33. doi: 10.1111/liv.14464 (PMC7384114; doi:10.1111/liv.14464)
Supplement: Supplementary file 1 — Supplementary Material [file LIV-40-1620-s001.pdf]

Figure S1. Related to Figure 1: Metabolic assessments

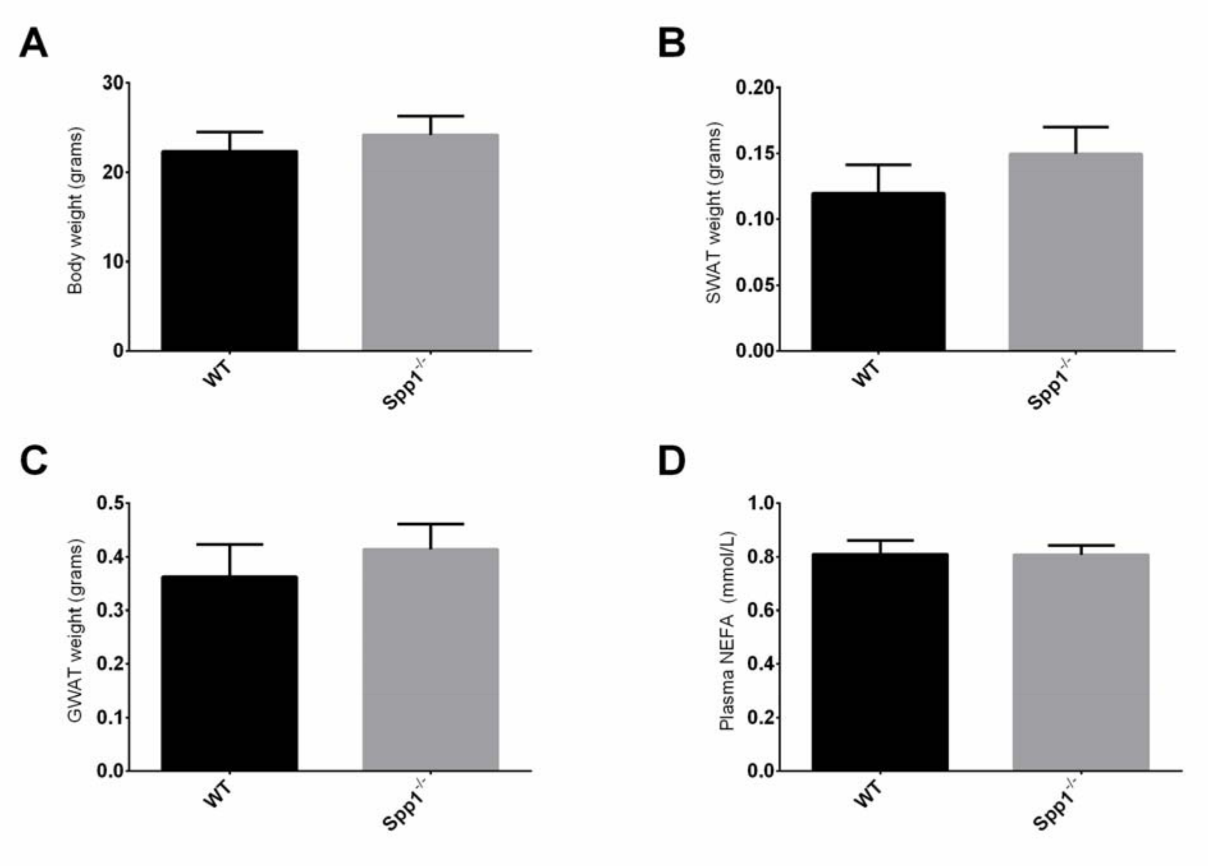

Figure S1. Related to Figure 1: Metabolic assessments. (A) Body weight, (B) subcutaneous and (C) gonadal white adipose tissue weight, expressed in grams. (D) Enzymatic quantification of plasma non-esterified fatty acids (NEFA). Tissues were harvested from 8-week-old mice (n = 8 per group).

Figure S2. Related to Figure 1 and Figure 2: Assessments in untreated, normal chow-fed mice and in mice acutely injected with streptozotocin.

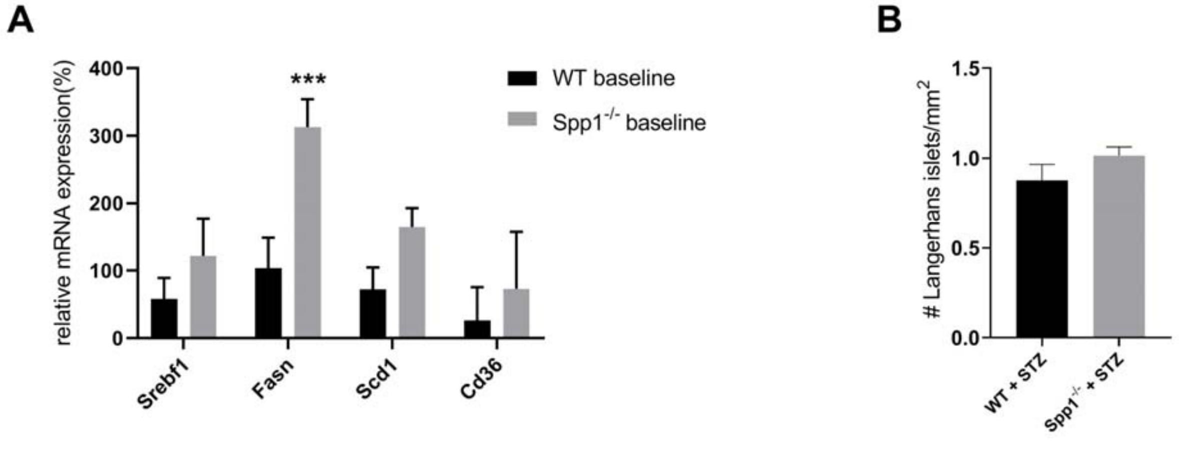

Figure S2. Related to Figure 1 and Figure 2: Assessments in untreated, normal chow-fed mice and in mice acutely injected with streptozotocin. (A) Gene expression anlyses of metabolic factors in untreated, normal chow-fed Wt and Spp1-/- mice. (B) Amount of intact pancreatic Langerhans islets, manually counted on H&E-stained, scanned sections (n = 8 per group).

Figure S3. Related to Figure 8: Hepatic phenotype of drop out mice

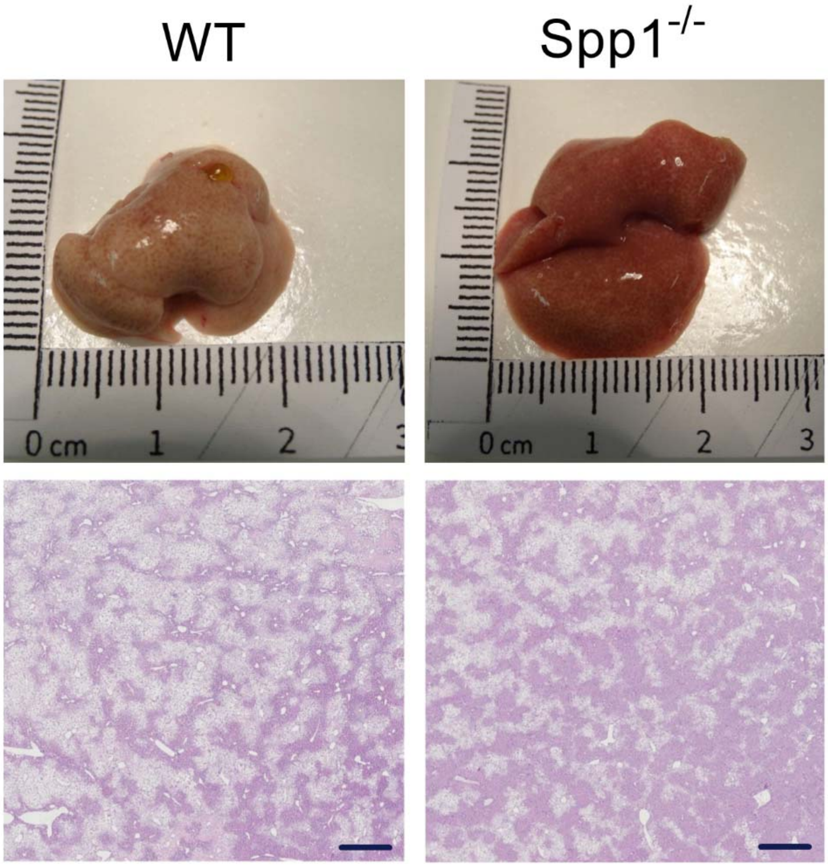

Figure S3. Related to Figure 7: Hepatic phenotype of drop out mice. Gross- and histological representation of drop-out livers. Scale bar = 1 mm. Tissues were harvested between the 11th and the 19th experimental week.
